# Micro- and nanoplastic exposure: male reproductive toxicity, underlying mechanisms, and research prospects

**DOI:** 10.3389/fendo.2026.1940055

**Published:** 2026-10-19

**Authors:** Min Wang, Jie Zheng, Caomeihui Shen, Chunxiao Liu, Shuai Ma, Yixin Wang, Minghui Fan, Minjia Sheng

**Affiliations:** 1 The Reproductive Medical Center, China-Japan Union Hospital of Jilin University, Changchun, China; 2 Jilin Provincial Key Laboratory of Reproductive Biology, Changchun, China; 3 College of Animal Science, Jilin University, Changchun, China; 4 Norman Bethune Third School of Clinical Medicine, Jilin University, China-Japan Union Hospital of Jilin University, Changchun, China; 5 Department of Obstetrics and Gynecology, The Second Hospital of Jilin University, Changchun, China; 6 Guanghua Academy, Shanghai, China

**Keywords:** endocrine disruptors, environmental exposure, infertility, male, microplastics, nanoplastics, reproductive health

## Abstract

Diagnosis and treatment of male infertility present persistent clinical challenges, with etiologies spanning genetic anomalies, endocrine disturbances, genital tract infections, varicocele, lifestyle factors, and environmental exposures. Among these, exposure to environmental contaminants is increasingly recognized as a critical contributor to male reproductive impairment and declining sperm quality. Micro- and nanoplastics (MNPs), as ubiquitous emerging environmental pollutants, can enter the human body via three primary routes: gastrointestinal ingestion, respiratory inhalation, and dermal contact. Upon internalization, MNPs trigger male reproductive toxicity through diverse molecular pathways, including the induction of oxidative stress, DNA damage, disruption of sex hormone secretion, energy metabolism dysfunction, gut microbiota dysbiosis, ferroptosis, and aberrant autophagy. Cumulative evidence suggests that these mechanisms may act in concert to compromise sperm quality and male fertility; furthermore, such exposure has been associated with an elevated risk of developmental abnormalities in offspring in certain experimental models. In this review, we systematically elucidate the mechanisms linking MNP exposure to the pathogenesis and progression of male infertility. We also aim to offer novel insights for the clinical diagnosis and management of male infertility, provide theoretical references for preconception care and reproductive health, and establish a scientific foundation for advancing targeted prevention and control strategies against MNP pollution.

## 1 Introduction

Global prevalence of male infertility has been increasing annually, emerging as a critical public health challenge worldwide (1). Male infertility is a multifactorial condition arising from the interplay of diverse pathological factors, including sexual dysfunction, hypogonadism, and spermatogenesis impairment. In parallel, the large-scale production and widespread application of plastic-derived consumer products have brought considerable convenience to daily life. Microplastics (MPs), a primary category of persistent plastic pollutants, have been extensively utilized owing to their unique physicochemical properties, such as light weight, high mechanical strength, excellent plasticity, and superior portability. Today, plastic-based components are ubiquitous in daily consumer goods, ranging from packaging materials and personal care products to household items and electronic devices. Epidemiological and environmental surveys indicate that over 80% of plastic products are discharged into the natural environment without undergoing effective standardized treatment, and subsequently fragment into MPs with diameters of <5 mm under long-term physical abrasion, photochemical degradation, and microbial decomposition. Since the term MPs was first formally proposed by Thompson et al. in 2004, research on this class of emerging environmental pollutants has received extensive global attention (2). MPs pose severe threats to global ecological security due to their extremely high environmental persistence and can enter the human body through three dominant exposure routes: gastrointestinal ingestion, respiratory inhalation, and dermal contact. Once absorbed into the systemic circulation, these particles may accumulate in certain tissues; however, the potential for subsequent harm to human organs remains incompletely understood and varies considerably with particle type, size, dose, exposure route, and the experimental model or species examined. A growing number of human biomonitoring studies have confirmed the detectable presence of MPs in various human tissues, including the gastrointestinal tract, lung, feces, blood, breast milk, and even ovarian follicular fluid (3–5). Of particular note, recent groundbreaking studies have successfully identified MPs in human testicular tissue, with an average concentration of 329.44 μg/g; among all polymer types identified, polyethylene (PE) is the most prevalent. Moreover, in a recent study quantifying MPs in canine and human testes, the concentration in testicular tissue was considerably higher than that reported in other visceral organs such as the liver and lung, suggesting a potential tissue-specific accumulation pattern in the male reproductive system under the tested conditions (6). Nanoplastics (NPs), plastic particles smaller than 100 nm, have also been shown to cause progressive male reproductive dysfunction in mammalian experimental models (7). Given that MPs can further fragment into NPs in the internal environment, these findings have raised public concerns regarding the potential reproductive toxicity of MPs and their smaller derivatives. This compelling scientific evidence further highlights the urgent necessity for in-depth research on the reproductive hazards of MPs and NPs. These observations underscore the urgent need for in-depth investigation into the reproductive hazards of MPs and NPs. Accordingly, systematically deciphering the molecular mechanisms underlying reproductive system injury induced by micro- and nanoplastics (MNPs), and developing targeted prevention and intervention strategies, is of great significance for safeguarding long-term reproductive health in humans and their offspring, although direct clinical evidence in humans remains limited.

A systematic and comprehensive literature search was performed using PubMed, Web of Science Core Collection, and Scopus, capturing all eligible publications up to December 2025. The search syntax combined Medical Subject Headings and free-text terms relevant to MPs, NPs, reproductive health, endocrine disruptors, and environmental exposure, testis, sperm, infertility, etc. Peer-reviewed original research articles and authoritative reviews published in English were considered eligible, whereas non-peer-reviewed preprints, conference abstracts, editorials, case reports, and non-English publications were excluded. Two independent reviewers screened titles and abstracts to eliminate duplicate and irrelevant records. We included studies addressing environmental exposure characterization, human biomonitoring, *in vivo* mammalian toxicopathology, *in vitro* cellular and molecular mechanisms, intergenerational toxicity, and therapeutic or preventive strategies. Studies lacking essential physicochemical particle characterization, presenting duplicate datasets, or demonstrating inadequate methodological rigor were systematically excluded to ensure data reliability. It is noteworthy, however, that the experimental conditions employed in the reviewed studies often differ substantially from realistic human exposure settings.

## 2 Male infertility

### 2.1 The epidemiological status of male infertility

Male infertility represents a prevalent disease in andrology and reproductive clinics. It is clinically defined as the failure of conception attributable to male factors in fertile couples who maintain regular unprotected sexual intercourse for more than 12 consecutive months (8). This disease possesses intricate pathogenic mechanisms stemming from multiple etiological factors, including genetic abnormalities, endocrine disorders, genital tract infections, varicocele, immune abnormalities, unhealthy lifestyles, and environmental exposure (9, 10). Exposure to environmental hazardous substances such as chemical pollutants and radiation, as well as various occupational hazards, continuously threatens male reproductive health. Sperm quantity and quality serve as core indicators for evaluating male reproductive function. The process of spermatogenesis is regulated by a variety of regulatory factors, and dysfunction in any link of this process can trigger spermatogenic disorders and further impair male fertility. Meta-analysis evidence reveals that global male sperm counts have exhibited a significant downward trend from 1973 to 2018, with a more drastic decline observed after 2000 (11, 12). Internal pathogenic factors of male infertility, including chromosomal abnormalities, gene mutations, and endocrine dyscrasia, adversely affect the development of the male reproductive system and spermatogenesis. Meanwhile, external risk factors such as smoking, alcohol consumption, and sleep deprivation can also induce damage to the male reproductive system.

### 2.2 Diagnostic and treatment bottlenecks

Current diagnostic and therapeutic strategies for male infertility have inherent limitations in clinical practice. Routine semen analysis cannot fully evaluate comprehensive sperm quality, endocrine examination results exhibit unstable fluctuations, and imaging examinations fail to precisely identify subtle reproductive pathological lesions. In terms of clinical treatment, pharmacotherapy, surgical intervention, and assisted reproductive technology can achieve certain therapeutic effects but still have undeniable drawbacks. Pharmacological treatment efficacy varies considerably among individual patients, and long-term medication intervention commonly reduces patient treatment compliance. The therapeutic success rates of conventional surgeries, including varicocelectomy and seminal tract recanalization surgery, are affected by multiple confounding factors, thereby leading to unstable clinical outcomes. Although assisted reproductive technology provides effective therapeutic options for infertile males, *in vitro* fertilization and embryo transfer and intracytoplasmic sperm injection still carry potential risks of passing hereditary defects to offspring. Additionally, female age and physical status significantly affect the overall success rate of assisted reproductive technology treatment (13). Refractory male infertility imposes severe psychological pressure on male patients, undermines family happiness and harmony, and further poses a serious threat to public health. In this context, exploring safer, more efficacious, and economical alternative or adjuvant therapies is urgently necessary for clinical practice. Accumulating studies confirm that environmental pollution-induced pathological changes, including endocrine disruption, reactive oxygen species (ROS)-mediated sperm DNA damage, and genetic and epigenetic abnormalities, are closely associated with the occurrence of male infertility (14, 15). New evidence suggests that exposure to MPs may be a new risk factor for the occurrence and progression of male infertility. Therefore, investigating the biological effects and molecular mechanisms of MPs in male infertility provides valuable theoretical and clinical implications for male reproductive health protection.

## 3 MNPs

### 3.1 Sources and distribution of MNPs

The term “MPs” was first introduced by Thompson et al. in 2004, and MPs are now widely defined as synthetic polymer particles with a diameter of <5 mm, whereas NPs are generally defined in environmental toxicology as plastic particles with dimensions below 1 µm (2). Researchers classify MPs into primary and secondary MPs based on their sources. Primary MPs mainly originate from microbeads in personal care products, synthetic textile fibers, and industrial raw materials. In contrast, secondary MPs derive from the fragmentation of large plastic wastes through photo-oxidation, physical abrasion, and microbial decomposition, and they exhibit greater heterogeneity in physicochemical characteristics (16). Common polymer components of MPs include PE, polypropylene (PP), polystyrene (PS), polyvinyl chloride (PVC), polyethylene terephthalate (PET), polyamide (PA), and polymethyl methacrylate (PMMA), which present diverse morphological types, including particles, fragments, fibers, films, and spheres. MPs are ubiquitously distributed across multiple environmental media, including oceans, freshwater, soil, and atmosphere, and also present in daily consumer products such as drinking water, food, cosmetics, clothing, and even automobile emissions. Global plastic pollution continues to escalate, and the total amount of environmental plastic waste is projected to reach 12 billion tons by 2050 (17). MPs have spread across nearly all global environmental matrices, with residues detected even in polar regions (18). River input serves as the major source of marine MPs, and MPs also occur widely in lakes and other freshwater systems. Atmospheric MPs predominantly consist of synthetic fibers, with higher deposition levels in urban areas than in suburban areas. Atmospheric deposition contributes to soil MP contamination, and industrial areas exhibit extremely high soil MP loads (19, 20). MP contamination is commonly detected in various daily foods, such as seafood, fruits and vegetables, table salt, beer, dairy products, and bottled water (21, 22). MPs in soil can be absorbed by crop roots, and environmental MPs can enter the human diet through multiple trophic levels, contributing to overall dietary exposure. The actual risk, however, varies with MP type, concentration, and the specific food chain involved. NPs have also been detected in environmental samples and are increasingly recognized as a critical component of plastic pollution due to their higher penetration potential and distinct toxicological profiles.

### 3.2 Physical and chemical properties of MNPs

MPs exhibit a broad particle size range and diverse morphologies, with predominant chemical compositions including PE, PP, PS, and PET. These particles are physicochemically stable and can persist in the environment for over a century (23). Their high specific surface area and strong hydrophobicity are core risk characteristics. Adsorption capacity increases with specific surface area and hydrophobicity, enabling MPs to adsorb heavy metals, polycyclic aromatic hydrocarbons, pesticides, and plastic additives via hydrophobic interactions, electrostatic forces, and hydrogen bonding, forming composite pollutants that induce synergistic toxicity (24–26). Environmental factors such as illumination, temperature, pH, and oxygen availability drive MP aging, which reduces particle size, increases surface roughness, decreases hydrophobicity, and alters MP migration and accumulation. Owing to their small size, strong adsorption, and non-biodegradability, MPs readily carry environmental pollutants into the human body through direct exposure or food chain transmission (27). When MNPs enter environmental or biological fluids, they acquire a layer of adsorbed organic matter, proteins, and biomolecules, forming an “eco-corona” or “biomolecule-corona.” This corona alters surface charge, hydrophobicity, and aggregation state, thereby modulating MNP transport, cellular recognition, and uptake efficiency (28). As demonstrated in the context of other internal biological barriers, corona formation can critically influence nanoparticle translocation across endothelial and epithelial layers (29, 30). Extending these principles to the male reproductive system, corona-coated MNPs may exhibit altered penetration across the Blood-Testis Barrier compared with pristine particles. NPs exhibit higher specific surface area, greater surface reactivity, and enhanced biological barrier penetration compared with MPs, which may result in distinct toxicokinetic profiles and more severe biological effects. It should be noted that most toxicological studies have employed PS as a reference polymer, whereas systematic comparative data on PE, PP, PET, and PVC remain limited. Direct cross-polymer comparisons are therefore currently difficult to establish, and future studies are warranted under standardized conditions.

### 3.3 Exposure pathways of MNPs

Human MP exposure mainly occurs through three pathways: gastrointestinal ingestion, respiratory inhalation, and dermal contact, among which gastrointestinal ingestion and respiratory inhalation are the dominant routes (31). Gastrointestinal ingestion contributes the largest exposure proportion and primarily originates from contaminated food. Adults exhibit a weekly MP exposure load of 0.1–5 g, while infants using plastic feeding bottles present a substantially higher daily MP intake than adults. MPs are widely present in water bodies, and bottled water contains considerable MP residues with an average abundance of 325 particles per liter and a maximum detected concentration of 10, 000 particles per liter (32, 33). Welle et al. also detected MPs in beer and honey, further confirming that dietary ingestion acts as a critical route for human MP exposure. Globally, up to 94% of commercial salt products contain detectable MP contaminants (34). Atmospheric MPs primarily derive from the washing of synthetic textiles, the shedding of construction materials, the abrasion of plastic products, and waste incineration and landfilling, from which they enter the human body via respiratory inhalation. Relevant studies have shown that workers engaged in textile and chemical industries face significantly higher inhalation risks, and long-term atmospheric MP inhalation can trigger airway and pulmonary inflammation. Dermal MP exposure mainly stems from daily skincare and cosmetic products (35). NPs, defined as plastic particles smaller than 1 µm, can penetrate the human skin barrier, with enhanced permeability in aged skin and pore-containing areas. Specifically, particles smaller than 40 nm can penetrate Langerhans cells surrounding hair follicles (36). Nevertheless, current research still lacks direct evidence for human tissue damage caused by dermal MP exposure (37). Iatrogenic MPs, a special subtype of MPs, can directly enter the human circulatory system through invasive medical procedures (38). Existing studies confirm that disposable medical devices continuously shed and release large quantities of MPs into the human body or the surrounding environment during use and disposal procedures, and such intensive MP release markedly elevates human exposure risks (39) (**Figure 1**). While these findings highlight potential health risks, it should be noted that the exposure doses and particle characteristics used in laboratory studies may not directly correspond to human environmental exposure.

**Figure 1:**
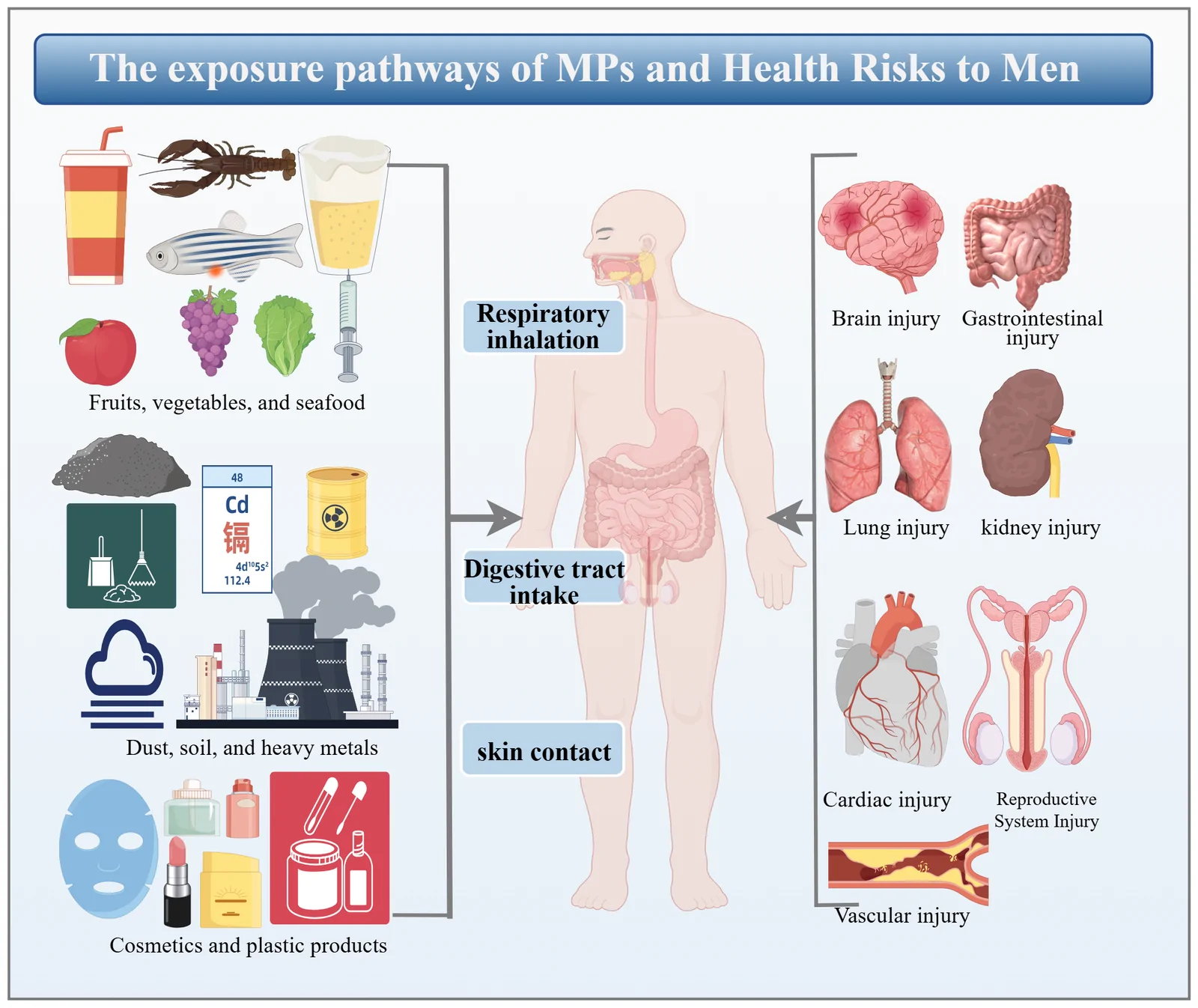
The main pathways of human exposure to MPs and their impact on health. MPs enter the human body through inhalation via the respiratory tract, ingestion through the digestive tract, and skin contact, causing damage to multiple organs, including the brain, gastrointestinal tract, lungs, kidneys, heart, blood vessels, and the male reproductive system.

### 3.4 Bioaccumulation of MNPs

As emerging environmental pollutants, MPs attract extensive research attention regarding their *in vivo* distribution, metabolism, and excretion characteristics (40). MPs possess the capacity to cross biological barriers and subsequently distribute and accumulate in multiple organ systems. After entering the human body, MPs penetrate intestinal and pulmonary barriers to access the bloodstream and accumulate in the digestive, respiratory, urinary, reproductive, and circulatory systems, with fine particles being able to migrate throughout systemic tissues. Studies have detected MPs in human peripheral blood, with an average concentration of 1.6 μg/mL (41), and MP residues have also been found in human thrombi (42). Humans primarily eliminate MPs through fecal and urinary excretion, with fecal discharge serving as the predominant clearance pathway. The detection of MPs in urine further confirms the metabolic elimination of MPs via the urinary system (43, 44). Plastic degradation generally includes physical, chemical, and biological approaches, all of which exhibit inherent limitations. Physical degradation fails to completely remove plastic particles, chemical degradation easily induces secondary pollution, and biological degradation faces severe practical implementation barriers (45). MPs retained *in vivo* can carry drug-resistant bacteria and persistent toxic substances due to their strong adsorption capacity and continuously exert toxic effects, thereby exacerbating cumulative damage to human physiological functions and health (46). Notably, NPs, due to their smaller size and higher surface reactivity, exhibit greater potential than MPs to cross biological barriers, accumulate in deeper tissues, and induce more pronounced toxic responses. However, quantitative data on NP bioaccumulation in humans remain scarce.

### 3.5 Health risks faced by MNPs

MPs can penetrate cell membranes and enter lysosomes and subsequently accumulate in mitochondria, the endoplasmic reticulum, and cell nuclei. The internalized MPs alter the structure and function of organelles, inhibit cell growth, and trigger a series of toxic responses via multiple mechanisms, including oxidative stress, energy metabolism disorder, and gut microbiota dysbiosis (47). The severity of toxicity induced by MPs primarily depends on particle size. Smaller plastic particles possess stronger adsorption capacity, a larger relative specific surface area, and can release higher levels of chemical components, thereby causing more severe adverse biological effects (48). Additionally, MPs frequently combine with exogenous substances to form mixed pollutants, particularly endocrine-disrupting chemicals such as bisphenol A and phthalates, which further enhance the combined toxic effects of contaminants. MPs can deposit and accumulate within the male reproductive system. In one study, Hu et al. administered fluorescent-labeled polystyrene nanoplastics (PS-NPs) to rats for one week and detected obvious fluorescent signals in frozen sections of rat testicular tissues, confirming that PS-NPs can penetrate the digestive tract and ultimately accumulate in testicular tissues (49). Consistent with these findings, Wei et al. demonstrated that exposure to polystyrene MPs (PS-MPs) significantly reduces the counts of viable epididymal sperm and spermatogenic cells, while markedly increasing the sperm malformation rate in male rats (50). Collectively, these findings underscore that exploring the adverse effects and underlying molecular mechanisms of MNP exposure on male infertility holds profound practical and clinical significance. Given the limited direct clinical evidence in humans, the translation of these experimental findings to human health risk assessment should be made with caution.

## 4 The impact of MNP exposure on male reproductive health

### 4.1 Experimental Evidence from animal models

A substantial body of experimental evidence from rodent models has demonstrated that MNPs can directly induce testicular injury, oxidative stress, and sperm quality deterioration under controlled exposure conditions. The testis serves as a core organ of the male reproductive system and undertakes critical endocrine and spermatogenic functions. In rodent models, MP exposure has been shown to induce testicular histological alterations, spermatogenic disorders, and hormonal secretion disturbances. Multiple studies have confirmed that MP exposure damages testicular tissues, reduces testicular organ index, elevates cellular apoptosis levels, and triggers structural damage, including outer wall rupture and vacuolization of seminiferous tubules (51). MP-induced spermatogenic abnormalities in mice manifest as loosening and vacuolization of the spermatogenic cell layer, depletion of spermatogenic cells, and increased apoptotic rates. Germ cells at all developmental stages exhibit atrophy, shedding, and apoptosis, accompanied by significant reductions in sperm count and motility as well as a sharp rise in sperm malformation rate (52–54). Small-sized plastic particles (<10 μm) have been shown to cross the epithelial barriers of the alveoli and digestive tract, enter the lymph and blood circulation, and finally accumulate in testicular tissues, where they trigger oxidative stress, inflammatory responses, and cellular apoptosis. After intragastric administration of PS-MPs, mice exhibit obvious declines in sperm quality and serum testosterone levels, along with disordered arrangement and extensive shedding of spermatogenic cells (55). PS-MP exposure also causes severe spermatogenic cell damage, presenting as vacuolization, atrophy, and even exfoliation of germ cells within seminiferous tubules (56). These findings strongly demonstrate that single MP exposure exerts detrimental impacts on testicular structure and sperm function, thereby inducing prominent male reproductive toxicity.

In addition to single exposures, combined exposure experiments have further elucidated the synergistic toxic effects of MNPs with other environmental contaminants. Environmental MPs propagate through bioaccumulation and food chain transmission, ultimately posing severe threats to human health. Under real environmental conditions, organisms are frequently exposed to a mixture of contaminants rather than a single pollutant. Due to their small particle size, strong adsorption capacity, and non-biodegradable properties, MPs readily bind with other environmental pollutants and facilitate contaminant bioaccumulation *in vivo* (57, 58). Heavy metals represent ubiquitous and persistent environmental pollutants, and MP–heavy metal complexes have been shown to impair organism health through food chain transfer (59). Lan et al. revealed that the synergistic interaction between MPs and cadmium suppresses the expression of the Keap1-Nrf2 signaling pathway and its downstream genes, induces lipid peroxidation and ferroptosis, and ultimately causes structural and functional abnormalities in reproductive tissues (60). The co-exposure of MPs and sodium fluoride has also been demonstrated to trigger synergistic reproductive toxicity in mammals, manifested as testicular structural defects, impaired spermatogenesis, and increased testicular cell apoptosis (61). Combined exposure to MPs and heavy metals significantly amplifies reproductive toxic effects compared with single exposure (62). Existing studies have shown that PS-MPs affect the viability of mouse spermatocytes, and co-exposure to MPs and cadmium exacerbates testicular tissue injury, reduces sperm quality and testosterone secretion, and induces testicular damage by interfering with the ferroptosis signaling pathway (63). In addition to heavy metals, MPs have been found to generate combined toxic effects with other environmental contaminants in the reproductive system. Co-exposure to microcystins and PS-MPs aggravates testicular structural damage, elevates cellular apoptosis, deteriorates sperm quality, and disrupts testosterone synthesis in mice (64, 65). These experimental findings demonstrate that MNP exposure exerts detrimental impacts on testicular structure and sperm function in animal models, thereby inducing prominent male reproductive toxicity under the tested conditions.

### 4.2 Human biomonitoring and epidemiological evidence

Human biomonitoring and epidemiological studies have provided preliminary evidence linking MNP exposure to adverse reproductive outcomes, although these data remain limited and largely correlational. Researchers have detected MPs in multiple human male biological specimens. Clinical observational studies have reported that the MP concentration in human semen can reach 15.34 μg·mL^-^¹, which exceeds the corresponding concentration in human blood. Seminal MPs present diverse morphologies, including fibers, fragments, and films, with particle sizes ranging from 21.76 μm to 286.71 μm (66). Montano et al. identified eight types of MNPs with particle sizes of 2–6 μm in human semen samples, among which PS, PE, and PP dominate the detected plastic components (67). Yang et al. further detected multiple MNP subtypes in human semen, testicular tissues, and percutaneous puncture fluid of epididymal cysts (68). A meta-analysis focusing on MP-induced sperm quality impairment demonstrated that individual exposure to MPs or NPs significantly reduces sperm concentration by 5.99 million per milliliter, increases sperm abnormality rate by 10.65%, and decreases sperm motility by 14.62% (69). Codrington et al. firstly confirmed the presence of MPs in human penile tissues. Although the detected MP abundance is relatively low, all positive samples derive from patients with erectile dysfunction, suggesting a potential correlation between MP exposure and the occurrence of male sexual dysfunction (70). Given that the penis is a highly vascularized organ, the presence of MPs in penile tissue raises the possibility of vascular-mediated pathology. Although Codrington et al. did not directly examine functional consequences, accumulating evidence from experimental models indicates that MNP exposure can induce endothelial dysfunction through oxidative stress and inflammatory responses, which are known to reduce nitric oxide bioavailability and impair vasodilation (71, 72). Furthermore, MP-induced microvascular damage and disruption of vascular integrity have been observed in other organ systems (42), suggesting that similar mechanisms may operate in penile vasculature. However, direct evidence linking MP accumulation in penile tissue to ED pathophysiology remains to be established, and this proposed connection should be considered speculative until confirmed by mechanistic studies in relevant vascular or erectile tissue models. In humans, polytetrafluoroethylene detection in semen has been found to correlate significantly with deteriorated semen quality, characterized by decreased total sperm count, sperm concentration, and progressive motility, which reveals the potential reproductive hazards of MP exposure (73). It is important to note that these human findings are primarily observational and do not establish causation. The studies reported to date have generally involved small sample sizes, limited population coverage, and potential confounding factors that have not been fully addressed. While these correlational data provide valuable clues for potential health risks, direct clinical evidence validating that MNPs cause male infertility in humans remains insufficient (**Table 1**).

**Table 1:** The impact of MNPs on the male reproductive system.

Research object	Detection substances	Particle size (μ m)	Main results	References
Human	MNPs	2-6 μ m	Eight types of MNPs were detected in semen, with the top three being PS, PE, and PP	(67)
Human	MNPs	0.72-7.02 μ m	Identification of eight different MNPs in semen, PS, and PVC affecting sperm motility	(66)
Human	MNPs	–	MNPs were identified in the testes, mainly PE, with an average abundance of 329.44 μ g/g	(6)
Human	MNPs	20-100nm	The average abundance of MNPs in testes was (11.60 ± 15.52) particles/g, and in semen it was (0.23 ± 0.45) particles/mL	(117)
Human	MNPs	–	Tested for PE, PVC, and PA in the testicles; semen detection revealed PS, PVC, PA, PMMA; epididymal cyst only found PVC	(68)
Mouse	PS-MPs	0.5, 4, 10 μ m	Testicular histological changes, abnormal sperm, decreased testosterone, mediating the LH receptor/cAMP/PKA/StAR pathway	(118)
Mouse	PS-MPs	0.5, 4, 10 μ m	Decreased sperm quality and testosterone, induced inflammation, disruption of the blood-testis barrier, and shedding of spermatogenic cells	(88)
Mouse	PS-MPs	5 μ m	Serum testosterone significantly decreased in a dose-dependent manner	(50)
Mouse	PS-MPs	5-5.9 μ m	Decreased sperm count and motility, increased deformity rate, decreased metabolic enzyme activity, increased oxidative stress levels, activation of JNK and p38-MAPK	(71)
Mouse	PS-MPs	5 μ m	Disruption of oxidative stress levels, decreased sperm quality, impaired blood-testis barrier, activation of autophagy-related pathways	(62)
Mouse	PS-MPs	4-10 μ m	Elevated levels of ROS, increased mTORC1 activity, decreased mTORC2 activity, disruption of blood-testis barrier integrity, and impaired sperm production	(119)
Mouse	PS-NPs	0.5 μ m	Oxidative stress occurs, the p38MAPK pathway is activated, blood-testis barrier integrity is disrupted, spermatogenic cell apoptosis occurs, and sperm quality decreases	(120)
Mouse	PS-MPs	10 μ m	Decreased antioxidant enzyme activity, decreased plasma testosterone levels, decreased sperm quality, testicular damage, and reduced number of germ cells	(81)
Mouse	PS-MPs	5 μ m	Increased autophagy and apoptosis, reduced steroidogenesis in Leydig cells	(121)
Mouse	PS-NPs	25, 50nm	Decreased serum testosterone concentration and downregulation of related gene expression	(122)
Mouse	PS-NPs	50, 100nm	Oxidative stress occurs, leading to DNA breakage and decreased mitochondrial activity, as well as increased acrosomal damage	(77)
Mouse	PS-MPs	10 μ m	The number and vitality of sperm decrease, and germ cells in the seminiferous tubules undergo vacuolization, atrophy, and even shedding	(55)
Mouse	PS-MPs	50, 90nm	Spermatogenesis is affected, and spermatogenic cells are damaged	(56)
Rat	PS-MPs	–	Sperm motility, quantity, and serum testosterone levels significantly decreased, and testicular structure changed	(123)
Mouse	PS-MPs	25-30nm, 1-5μ m	Gradual, linear, and linear velocities of sperm movement decrease	(53)
Mouse	PS-MPs	5μ m	Decreased epididymal sperm, increased malformation rate, testicular atrophy and apoptosis, increased NF - κ B, IL-1β, IL-6, and decreased Nrf2/HO-1	(72)
Gallus gallus	PS-MPs	5μ m	Epididymitis infiltration and interstitial bleeding, testicular tissue damage, and disruption of blood-testis barrier integrity	(99)
Zebrafish	MNPs, -MBC	50nm	Sperm destruction occurs in a dose-dependent manner	(57)
Zebrafish	PS-MPs, TCS	50nm	The increase in TCS levels in the testes exacerbates TCS-induced spermatogenesis	(80)
Mouse	PS-MPs, CdCI_2_	0.1, 1μ m	Testicular structure damage, reduced sperm quality, and testosterone levels	(63)
Mouse	PS-MPs, Cd	1μ m	Aggravated testicular structural damage, impaired spermatogenesis, and restricted sex hormone synthesis	(60)
Mouse	PS-MPs, NaF	5μ m	Aggravated testicular structural defects, impaired spermatogenesis, and increased apoptosis of testicular cells	(61)
Mouse	PS-MPs, MC-LR	1μ m	Exacerbating gonadal damage and reproductive endocrine disorders, the higher the concentration of MC-LR, the more pronounced its expression	(65)
Mouse	PS-MPs, MC-LR	4μ m	Disrupting testicular structure and increasing tissue apoptosis levels	(64)

MPs, microplastics; NPs, nanoplastics; MNPs, micro- and nanoplastics; PS, polystyrene; PE, polyethylene; PP, polypropylene; PVC, polyvinyl chloride; PA, polyamide; PMMA, polymethyl methacrylate; ROS, reactive oxygen species; MAPK, mitogen-activated protein kinase; JNK, c-Jun N-terminal kinase; NF-κB, nuclear factor kappa-B; IL-1β, interleukin-1 beta; IL-6, interleukin-6; Nrf2/HO-1, nuclear factor erythroid 2-related factor 2/heme oxygenase-1; mTORC1/2, mechanistic target of rapamycin complex 1/2; LH, luteinizing hormone; StAR, steroidogenic acute regulatory protein; TCS, triclosan; CdCl₂, cadmium chloride; Cd, cadmium; NaF, sodium fluoride; MC-LR, microcystin-LR; MBC, 4-methylbenzylidene camphor. Co-exposure conditions are indicated by a comma (e.g., “PS-MPs, CdCl₂” indicates combined exposure to polystyrene microplastics and cadmium chloride). Particle sizes are presented as reported in the original studies, with units of μm or nm as specified. “Human” refers to human biomonitoring studies; “Mouse, “ “Rat, “ “Gallus gallus, “ and “Zebrafish” refer to the corresponding animal models. Main results are summarized as reported by the cited authors.

## 5 Mechanism of MNP-induced male reproductive disorders

The mechanisms underlying MNP-induced male reproductive toxicity are not independent, discrete pathways but rather constitute a highly interconnected pathogenic network. Oxidative stress serves as the central hub of this network. Excessive ROS production triggers mitochondrial dysfunction and energy metabolism disorder, activates the Nrf2/MAPK/NF-κB signaling axes, and initiates inflammatory responses, ferroptosis, and apoptosis. Simultaneously, impaired autophagy amplifies cellular damage, while epigenetic changes and gut microbiota dysbiosis act as upstream modulators that influence the sensitivity and responsiveness of this network. The cumulative effect of these interconnected pathways ultimately leads to testicular damage, spermatogenic failure, hormonal imbalance, and intergenerational reproductive consequences. The following subsections detail each component of this integrated framework, with explicit cross-references to highlight their interconnections.

### 5.1 Epigenetic changes

Epigenetic regulatory mechanisms mainly include non-coding RNA modulation, DNA methylation, and histone modification. These epigenetic alterations function as upstream modulators that can both result from oxidative stress and regulate the expression of oxidative stress-related genes, establishing a bidirectional relationship with the central oxidative stress hub. Histone methylation serves as a potential mechanism mediating reproductive toxicity induced by MP exposure (74). DNA damage represents a core pathological mechanism of male infertility. MPs trigger DNA damage, disrupt sperm DNA integrity and chromatin structure, and induce significant alterations in sperm morphology and motility in a dose-dependent manner. *In vivo* mouse studies reveal that MP exposure significantly upregulates the expression of clk-2, cep-1, and egr-1 genes, and the overexpression of these genes closely correlates with DNA damage and subsequent germ cell apoptosis (75). MP-induced sperm DNA damage is heritable and can be transmitted to offspring (76). Compared with MPs, smaller-sized NPs exert stronger toxic effects, cause more severe damage to sperm organelles, and increase the incidence of sperm acrosome injury (77). In addition, MPs can induce sperm DNA damage in mice by regulating the PI3K/Akt signaling pathway and modulating SPARC expression (78). Of note, the PI3K/Akt pathway also intersects with the Nrf2 antioxidant response and autophagy regulation, further underscoring the integrated nature of these mechanisms.

### 5.2 Hypothalamic pituitary testicular axis disruption

The hypothalamic–pituitary–testicular (HPT) axis is essential for regulating systemic sex hormone homeostasis and maintaining normal male sexual function. Endocrine disruption is one of the pivotal mechanisms by which MPs impair male reproductive health, and this disruption is closely intertwined with oxidative stress, while hormonal imbalance further compromises antioxidant capacity, creating a vicious cycle. MPs significantly reduce serum levels of gonadotropin-releasing hormone, follicle-stimulating hormone, luteinizing hormone, and testosterone in mice, thereby disrupting testosterone homeostasis via HPT axis dysfunction (79). Co-exposure to PS-NPs and triclosan induces reproductive toxicity by disrupting the hypothalamic–pituitary–gonadal–hepatic axis in adult zebrafish and exacerbates triclosan-mediated spermatogenic suppression in male individuals (80). MPs suppress the activities of antioxidant enzymes in testicular tissues, trigger lipid peroxidation and ROS accumulation, and reduce plasma follicle-stimulating hormone and luteinizing hormone levels. Such alterations inhibit the LH-mediated LHR/cAMP/PKA/StAR signaling cascade, ultimately leading to prominent declines in intratesticular and circulating testosterone levels (81). MNPs activate the MAPK signaling pathway and the PI3K/AKT/mTOR pathway along with their downstream effector molecule 4E-BP1, while simultaneously interfering with the LHR-regulated cAMP/PKA signaling axis. These multi-targeted regulatory abnormalities jointly form a cascade inhibitory effect on StAR expression and further disturb testicular steroidogenesis (82). The MAPK pathway is also a key mediator of oxidative stress-induced testicular damage, illustrating the convergence of endocrine and oxidative pathways.

### 5.3 Oxidative stress as the central hub

Oxidative stress represents the central hub underlying MNP-triggered male reproductive toxicity, connecting all other mechanisms in an integrated network. MNP exposure disrupts the dynamic balance between ROS generation and the endogenous antioxidant defense system. This imbalance alters the activities and expression levels of antioxidant enzymes, including superoxide dismutase, catalase, and glutathione peroxidase, as well as the non-enzymatic antioxidant glutathione. Accumulated malondialdehyde, a terminal product of lipid peroxidation, further indicates disrupted redox homeostasis. Critically, ROS overproduction not only directly damages testicular cells but also activates downstream pathways, including the NF-κB pathway, mitochondrial dysfunction and energy deficit, and ferroptosis, while simultaneously being amplified by impaired autophagy. Xie et al. demonstrate that PS-MP exposure elevates ROS and malondialdehyde levels, reduces glutathione content, and activates the JNK and p38 MAPK signaling pathways in mouse testes, thereby deteriorating sperm quality and inhibiting testosterone synthesis (71). Li et al. report that oral PS-MP exposure significantly increases testicular ROS and malondialdehyde concentrations and downregulates the expression of catalase, superoxide dismutase, and glutathione peroxidase in mice. Such oxidative stress enhances p38 MAPK phosphorylation, suppresses the expression of blood–testis barrier (BTB) junction proteins and Nrf2, and decreases the Bcl-2/Bax ratio. These findings confirm that oxidative stress disrupts BTB integrity and triggers spermatogenic cell apoptosis via the MAPK-Nrf2 signaling cascade. Additionally, MPs aggravate oxidative damage by inhibiting the Keap1/Nrf2/HO-1 antioxidant pathway (83), and PS-MPs induce testicular premature aging through the Ca²^+^/ROS/NF-κB signaling axis (84). Notably, MNP-induced oxidative stress suppresses the synthesis of γ-aminobutyric acid and promotes neuronal apoptosis in the fetal brain, posing potential threats to embryonic neural development (85).

Three primary molecular mechanisms are responsible for MNP-mediated oxidative stress. First, metal oxide ingredients contained in plastic additives release free radicals after entering the human body and directly promote excessive ROS production; Second, MNP surfaces readily adsorb environmental pollutants, which further increase intracellular ROS burden and inhibit antioxidant enzyme activities after internalization. Third, MNPs activate inflammatory responses and upregulate pro-inflammatory cytokines such as TNF-α and IL-1β, which further stimulate ROS release and establish a positive “inflammation–ROS” feedback loop to exacerbate oxidative tissue damage.

### 5.4 Inflammatory response

Inflammatory responses induced by MNPs serve as one of the critical pathological processes that mediate male reproductive toxicity, and they are intimately linked to oxidative stress through a positive feedback loop — ROS activates NF-κB, which upregulates pro-inflammatory cytokines, and these cytokines further stimulate ROS production, creating a self-amplifying cycle. PS-MP directly stimulates peripheral blood monocytes to secrete cytokines and chemokines (86, 87). Jin et al. confirm that MPs penetrate the BTB and invade germ cells, while disrupting tight junctions between Sertoli cells. Such destructive effects significantly elevate the mRNA expression levels of pro-inflammatory factors, including TNF-α, IL-6, MCP-1, and CXCL10, thereby impairing BTB integrity, reducing sperm motility, and increasing sperm malformation rates (88). MPs modulate the Nrf2/HO-1/NF-κB signaling axis to upregulate pro-inflammatory mediators such as IL-1β and IL-6, while suppressing the expression of anti-inflammatory Nrf2/HO-1 molecules, which ultimately induces testicular inflammation and deteriorates sperm quality (72). The Nrf2 pathway, which plays a central role in antioxidant defense, also critically regulates this inflammatory response, highlighting the convergence of oxidative and inflammatory signaling at the Nrf2/NF-κB interface. Furthermore, crosstalk occurs between the ROS/MIF/NF-κB cascade triggered by M1 macrophage polarization and energy metabolism disorders, which synergistically drive the initiation and progression of testicular inflammation. The persistent chronic inflammatory state disrupts the immune microenvironment homeostasis of testicular germ cells and ultimately disturbs the entire developmental and maturation process of spermatogenesis.

### 5.5 Energy metabolism disorder

Energy metabolism disorder constitutes another vital mechanism underlying MNP-induced male reproductive toxicity, and it is both a cause and a consequence of oxidative stress. Mitochondrial dysfunction generates excessive ROS, while ROS further damages mitochondria, creating a vicious cycle. Long-term MNP exposure disrupts glucose metabolism, lipid metabolism, and mitochondrial function in mammals. Typical manifestations include decreased mitochondrial membrane potential, inhibited activities of mitochondrial electron transport chain complex I and complex IV, and reduced adenosine triphosphate production. The accompanying accumulation of ROS further exacerbates metabolic dysfunction. In addition, MNP exposure impairs PINK1/Parkin-mediated mitophagy, induces the accumulation of damaged mitochondria, and progressively aggravates cellular metabolic injury. This mitochondrial dysfunction not only fuels ROS production but also sensitizes cells to ferroptosis and apoptosis, demonstrating the interconnectivity of these pathways. In the male reproductive system, MNP exposure reduces sperm count by disrupting glucose metabolism and decreases serum testosterone levels by suppressing the activities of succinate dehydrogenase and lactate dehydrogenase, thereby compromising male reproductive function. Such toxic alterations are closely associated with the dysregulated expression of genes involved in glycolysis and lipid metabolism (89). Moreover, MNPs induce systemic protein catabolism to counteract pollutant stress, accompanied by decreased levels of total cholesterol and triglycerides, which indicates comprehensive metabolic dysfunction after MNP exposure. Collectively, MNPs disturb energy homeostasis through multiple regulatory pathways, which serve as a crucial contributor to MNP-triggered male reproductive toxicity.

### 5.6 Gut microbiota dysbiosis

Gut microbiota dysbiosis acts as a critical trigger that can initiate systemic oxidative stress and inflammation, rather than as an independent pathway. PS-NPs trigger male reproductive toxicity via crosstalk between gut microbiota and microbial metabolites (90, 91). PS-MP exposure reduces intestinal mucus secretion in mice, decreases the relative abundance of Firmicutes and Alphaproteobacteria, and ultimately impairs intestinal barrier integrity (92). MP exposure elevates the abundance of intestinal Prevotella, which exhibits a negative correlation with sperm motility. PS-MPs simultaneously increase the abundance of Bacteroides and Prevotella. The enriched pro-inflammatory bacteria drive Th17 cell translocation, upregulate the expression level of IL-17A, and activate downstream inflammatory cascades, eventually resulting in testicular tissue damage, spermatogenic dysfunction, and inhibited hormone synthesis. Fecal microbiota transplantation experiments conducted by Wen et al. further verify that the increased abundance of Bacteroides and Prevotella is positively correlated with testicular dysfunction (93). MP exposure also alters colonic microbial composition, reduces intestinal mucin secretion, and enhances amino acid metabolism, thereby triggering systemic immune responses characterized by decreased Th17 cell proportion and elevated serum inflammatory factors (94, 95). Notably, probiotic supplementation inhibits the PS-MP-activated IL-17A signaling pathway and alleviates gut microbiota-driven inflammatory injury, providing a promising intervention strategy for the prevention and treatment of MNP-induced male reproductive toxicity (96).

### 5.7 Ferroptosis

Ferroptosis plays a pivotal pathological role in male reproductive damage, and it represents an endpoint of oxidative stress, lipid peroxidation, and iron dyshomeostasis, mediated by the Nrf2-Keap1 and p38 MAPK pathways. Iron overload is recognized as one of the core mechanisms linking iron metabolic disorder with reproductive dysfunction. PS-MP exposure induces deteriorated sperm quality and testicular histological damage in male mice, accompanied by the downregulated protein expression of GPX4 and SLC7A11 as well as elevated lipid peroxidation levels, indicating the active involvement of ferroptosis in PS-MP-triggered male reproductive injury. Multiple signaling cascades are implicated in this pathological process, including Keap1-Nrf2 axis-mediated oxidative stress, NF-κB-driven inflammatory responses, and p38 MAPK pathway-dependent BTB disruption (97).

Exposure to PS induces testicular structural impairment and increased spermatogenic cell apoptosis. Transcriptomic analysis reveals that differentially expressed genes are significantly enriched in the p53 signaling pathway, accompanied by decreased Bcl-2 expression and upregulated levels of Bax, Caspase-3, and Caspase-9 (98). Hou et al. demonstrate that PS-MPs interfere with the Nrf2-Keap1 and Nrf2-NF-κB signaling axes to trigger excessive oxidative stress and inflammatory responses, upregulate pro-apoptotic proteins and downregulate anti-apoptotic mediators, and ultimately activate cellular apoptotic signaling (99). In addition, exposure to di(2-ethylhexyl) phthalate, a typical plasticizer, induces ferroptosis in mouse testicular cells via the HIF-1α/HO-1 pathway (100). Furthermore, the synergistic interaction between MPs and cadmium suppresses the Keap1-Nrf2 pathway and its downstream target genes, induces lipid peroxidation and ferroptosis, and eventually impairs male reproductive function (60). Thus, ferroptosis integrates oxidative stress, inflammatory signaling, and metabolic dysfunction into a final common pathway of cell death.

### 5.8 Autophagy abnormality

Abnormal autophagy represents a crucial molecular mechanism underlying MP-induced male reproductive toxicity, and it is intimately connected to mitochondrial dysfunction and ROS accumulation. Impaired autophagy fails to clear damaged mitochondria, leading to further ROS production and amplifying oxidative damage and apoptosis. Oral exposure to PS-NPs suppressed acrosomal autophagy in mice and promoted the ubiquitination and subsequent degradation of ras-related C3 botulinum toxin substrate 1 and cell division cycle 42, two Rho GTPases that critically regulate sperm morphogenesis and motility, thereby impairing sperm function and male fertility. Such molecular alterations trigger sperm structural defects and kinetic dysfunction, block sperm capacitation, and markedly reduce the success rate of *in vitro* fertilization. Additionally, NPs aggravate dibutyl phthalate-induced BTB dysfunction through autophagy inhibition. In testicular tissues, MNP exposure impairs male reproductive function via dual regulatory mechanisms of apoptosis and autophagy. MNPs not only induce spermatogenic cell apoptosis but also activate the PINK1/Parkin signaling pathway to trigger mitophagy, thereby causing irreversible spermatogenic cell damage. The PINK1/Parkin pathway links mitochondrial quality control with autophagic clearance, and its impairment exacerbates both oxidative stress and energy deficit. *In vitro* experiments further validate that NPs penetrate mouse spermatocyte GC-2spd cells and induce ROS-dependent mitochondrial apoptosis and excessive autophagy, which collectively disrupt normal spermatocyte physiological function.

### 5.9 Intergenerational transmission

Intergenerational transmission is best understood as a downstream consequence of the cumulative damage from the interconnected mechanisms described above, rather than as a separate parallel process. The oxidative stress, DNA damage, epigenetic alterations, and apoptosis triggered by MNP exposure in parental germ cells can be transmitted to offspring, affecting their reproductive development and function. Using a mouse model in which male mice were administered 200 nm PS-NPs at 2 mg/L via daily gavage for 35 days, Sun et al. reported significant intergenerational effects in the F1 offspring, including reduced body weight, testicular weight, and body length, along with testicular histological damage and decreased sperm count and motility (101). PS-MP exposure triggers testicular developmental disorders at postnatal day 35 and elicits spermatogenic dysfunction at PND70. RNA sequencing and bioinformatic analyses further demonstrate that hormone-mediated signaling pathways, G1/S transition of the mitotic cell cycle, co-regulation of androgen receptor activity, and the Hippo signaling pathway are critically involved in PS-MP-disrupted testicular development at the early postnatal stage (102). MP exposure not only impairs the generation and quality of sperm and oocytes but also penetrates the placental barrier to endanger embryonic development. The placenta serves as an essential protective barrier between mothers and fetuses. Garcia et al. detect abundant MPs in human placental tissues, with PE identified as the predominant component, which primarily presents as spherical or irregular particles ranging from 5 to 10 μm in size (103). Accumulating evidence confirms that MPs interfere with normal placental function and consequently induce adverse fetal developmental outcomes (104, 105). *In vitro* embryonic cell models further verify that cultivation in PS solution significantly elevates the metabolic and mitochondrial activity of human embryonic cells, indicating abnormal embryonic cellular responses triggered by MP stress (106).

Collectively, these mechanisms do not operate in isolation but rather form an integrated pathogenic network in which oxidative stress serves as the central hub. ROS overproduction triggers mitochondrial dysfunction and energy deficit, activates Nrf2/MAPK/NF-κB signaling axes, and initiates inflammatory responses, ferroptosis, and apoptosis, while impaired autophagy further amplifies cellular damage. Epigenetic changes and gut microbiota dysbiosis modulate the sensitivity of this network, and the cumulative effect of these interconnected pathways ultimately leads to testicular damage, spermatogenic failure, hormonal imbalance, and intergenerational reproductive consequences. This unified framework provides a comprehensive conceptual basis for understanding the complex pathogenesis of MNP-induced male reproductive toxicity and highlights potential nodes for future therapeutic intervention. However, the precise developmental window during which MNP exposure exerts its intergenerational effects, whether via direct interference with epigenetic reprogramming in primordial germ cells or via oxidative damage to mature sperm chromatin, remains to be determined and warrants future investigation.

## 6 Intervention measures

Current research on male reproductive toxicity induced by MNPs remains at an early stage, and no clinically validated targeted therapeutic interventions are currently available. The following strategies are categorized according to their evidence level (**Table 2**).

**Table 2:** Overview of intervention strategies for MNP-induced male reproductive toxicity, categorized by evidence type, target, reproductive benefit, human evidence, and current status.

Intervention strategy	Evidence type	Target	Evidence of reproductive benefit	Human evidence	Current status	Reference
Environmental source control						
Mangrove bioremediation	Environmental/field study	MP interception and degradation	Reduced MP abundance in aquatic environments	–	Implemented in pilot areas	(108)
Reed adsorption	Environmental/field study	MP uptake from water/sediment	Reduced environmental MP load	–	Under investigation	(124)
Specialized adsorbents	Laboratory study	MP removal from water/air	Reduced MP concentration in experimental systems	Limited	Under investigation	(125)
Daily behavioral modifications						
Steaming/boiling	Experimental study	Removal of MPs from aquatic products	Reduced MP content in food	Limited (laboratory only)	Recommended based on lab evidence	(112)
Boiled/filtered water	Observational/experimental	Avoidance of MP ingestion	Lower MP intake	Indirect evidence	Recommended	(126)
Natural fiber products	Experimental study	Reduced microfiber release	Decreased MP shedding	Limited	Recommended	(127)
Regular cleaning	Environmental study	Reduced indoor MP inhalation	Lower airborne MP exposure	–	Recommended	(128)
Occupational protective equipment	Observational/industrial	Reduced occupational MP exposure	Lower inhalation risk	Limited	Recommended	(129)
Pharmacological/nutritional interventions						
Chitosan supplementation	Animal study	Binding and excretion of intestinal MPs	Reduced MP accumulation; restored testicular function	None	Preclinical	(130)
Antioxidant agents	Animal study	Suppression of oxidative stress	Improved sperm quality; reduced testicular damage	None	Preclinical	(131)
Anti−inflammatory agents	Animal study	Suppression of inflammatory response	Reduced testicular inflammation; improved spermatogenesis	None	Preclinical	(132)

This table summarizes intervention strategies that have been proposed or tested for mitigating MNP-induced male reproductive toxicity. The strategies are organized into three major categories: (i) environmental source control (mangrove bioremediation, reed adsorption, specialized adsorbents), (ii) daily behavioral modifications (steaming/boiling, boiled/filtered water, natural fiber products, regular cleaning, occupational protective equipment), and (iii) pharmacological/nutritional interventions (chitosan supplementation, antioxidant agents, anti-inflammatory agents). For each strategy, the table provides the evidence type (environmental/field, laboratory, observational/experimental, or animal study), the primary target or mechanism, the evidence of reproductive benefit, the availability of human evidence (none, limited, or indirect), and the current translational status (implemented in pilot areas, under investigation, recommended based on lab evidence, or preclinical). It should be noted that no clinically validated intervention specifically targeting MNP-induced male infertility is currently available; all listed strategies remain at the environmental, behavioral, or preclinical level. MPs, microplastics; MNPs, micro- and nanoplastics. Dash (–) indicates no data or evidence available.

### 6.1 Macro-environmental source control technologies

From the perspective of environmental source control, natural bioremediation and artificial technical strategies can be adopted to mitigate MP/NP pollution at the macro-environmental level. Mangrove ecosystems have been shown to intercept and degrade aquatic MPs (107, 108), and reeds can adsorb and take up environmental MPs (109). In addition, various specialized adsorbents have been reported to effectively reduce MP abundance in water and atmospheric environments (110). These approaches target the reduction of environmental MP/NP loads at the source, thereby minimizing human exposure risks at the population level.

### 6.2 Targeted pharmacological interventions

Pharmacological or nutritional interventions have been explored in experimental models to alleviate MP-induced reproductive damage, although human evidence remains lacking. Chitosan has been reported to bind intestinal MPs and facilitate their excretion in animal models, suggesting a potential protective approach. Early intervention with antioxidants and anti-inflammatory agents has shown efficacy in suppressing excessive inflammatory responses and oxidative stress in rodent studies, thereby protecting male reproductive function under experimental conditions. However, these findings are derived from animal models with specific MP types, doses, and exposure regimens, and their translatability to humans requires further investigation.

### 6.3 Preconception care clinical strategies for andrology practices

At the individual and clinical level, daily behavioral and dietary modifications may help reduce MNP intake and mitigate potential reproductive risks, although direct evidence for improved fertility outcomes in humans remains limited. Aquatic products generally carry high MP loads; steaming and boiling exhibit superior efficacy in removing MPs compared with pickling and frying (111, 112). Bottled water contains higher MP concentrations than tap water; therefore, reducing the consumption of plastic-packaged beverages and plastic utensils, as well as prioritizing boiled or filtered drinking water, may help lower dietary MP exposure (113). For personal care products and textiles, avoiding commodities containing MP ingredients and preferring natural fiber products may reduce microfiber release during washing (114). Regular vacuuming and dust wiping can reduce the risk of inhalational MP exposure from indoor dust (115). For occupational populations in high-risk industries, standardized protective equipment is recommended to control occupational MP exposure, combined with regular health monitoring (116). These strategies may be considered as adjunctive recommendations in preconception care for couples planning pregnancy, although their clinical efficacy in preventing MNP-associated male infertility has not yet been validated. It is important to emphasize that all strategies described above, whether environmental, pharmacological, or behavioral, remain at the experimental or theoretical level. No clinically validated interventions specifically targeting MNP-induced male infertility have been established to date. Rigorous human clinical trials are urgently needed to evaluate the safety, efficacy, and feasibility of these interventions before they can be recommended for clinical practice.

## 7 Conclusion

As emerging environmental pollutants, MNPs exert toxic effects on the male reproductive system through multiple pathogenic mechanisms, including oxidative stress, DNA damage, hormonal secretion disturbance, energy metabolism dysfunction, and gut microbiota dysbiosis (**Figure 2**). These pathways exhibit synergistic crosstalk that may collectively trigger reproductive system injury, compromise sperm quality, impair fertility, and pose potential health risks to offspring in certain experimental models. Current studies on MNP-induced male reproductive toxicity have prominent limitations. Most investigations are based on rodent models and *in vitro* cell lines, limiting direct extrapolation to human physiology. Laboratory conditions often fail to simulate realistic human exposure scenarios, and most experiments employ single MNP exposure while neglecting combined exposure patterns of coexisting pollutants. Research predominantly focuses on acute and short-term effects, whereas bioaccumulative properties, chronic toxicity, intergenerational or transgenerational impacts, and precise molecular mechanisms remain insufficiently elucidated. Epidemiological studies in humans remain scarce, with available clinical investigations limited by small sample sizes, narrow population coverage, crude exposure assessment, and low detection accuracy. The absence of unified dose evaluation systems further hinders data comparison across studies. Direct clinical evidence validating the reproductive hazards of MNPs in humans and their combined toxicity mechanisms remains unclear. To address these gaps, future research should prioritize the following directions. First, a standardized MNP exposure dose evaluation system should be established, incorporating particle size distribution, surface modification, concentration ranges relevant to human exposure, and exposure duration, to enable meaningful cross-study comparison and risk assessment. Second, *in vitro* co-exposure matrices should be designed to better mimic realistic environmental conditions by incorporating environmentally relevant concentrations, mixtures of multiple polymer types, and coexisting pollutants that are commonly found in human exposure scenarios. Third, prospective cohort studies with robust exposure assessment and standardized outcome measures are urgently needed to bridge the gap between experimental findings and human health risk assessment. Additionally, the synergistic co-exposure of plastics and persistent organic pollutants on testicular histopathology warrants further investigation, as combined toxicity may exceed that of either class alone. Addressing these knowledge gaps will advance systematic understanding of MNP-mediated male reproductive damage and provide scientific evidence for clinical diagnosis and targeted treatment of male infertility.

**Figure 2:**
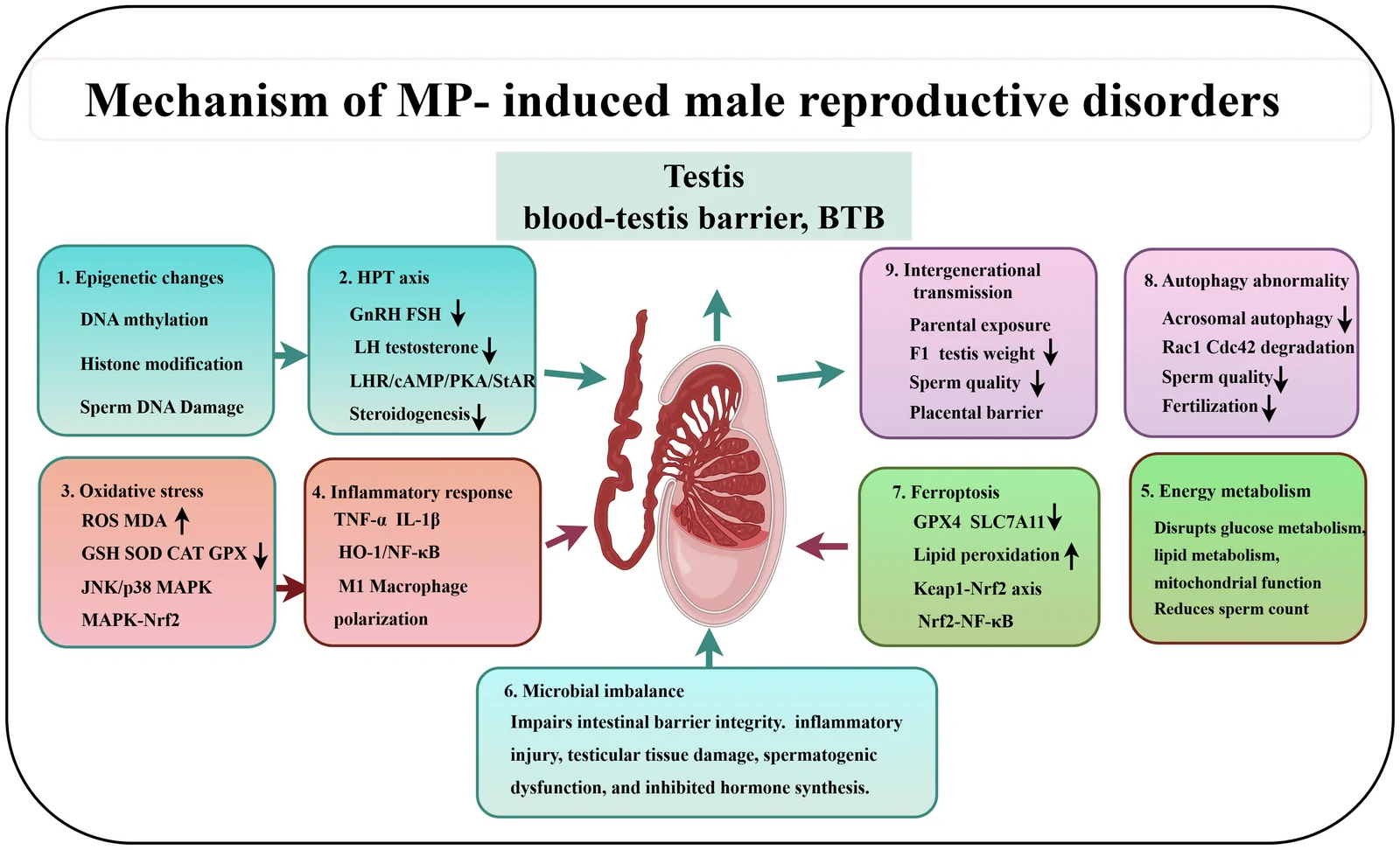
Mechanisms underlying MNP-induced male reproductive disorders. Solid boxes denote established mechanisms supported by direct experimental evidence. Dashed boxes indicate proposed mechanisms requiring further validation. Solid arrows indicate validated causal cascades; dashed arrows indicate putative crosstalk. Evidence sources are tagged as H (human studies), A (*in vivo* animal models), and C (*in vitro* cellular assays).
